# Neisseria cinerea in a Post-splenectomy Patient: A Rare Potentially Fatal Bacteremia

**DOI:** 10.7759/cureus.3007

**Published:** 2018-07-19

**Authors:** Ravikaran Patti, Sushilkumar S Gupta, Sharonlin Bhardwaj, Prameeta Jha, Arindam Ghatak, Yizhak Kupfer, Chanaka Seneviratne

**Affiliations:** 1 Internal Medicine, Maimonides Medical Center, Brooklyn, USA; 2 Critical Care, Maimonides Medical Center, Brooklyn, USA; 3 Internal Medicine, Maimonides, Brooklyn, USA; 4 Department of Critical Care Medicine, Maimonides Medical Center, Brooklyn, USA; 5 Pulmonary and Critical Care, Maimonides Medical Center, Brooklyn, USA

**Keywords:** neisseria cinerea, bacteremia, post splenectomy patient

## Abstract

Neisseria cinerea is a commensal which usually resides in the human respiratory tract. Very rarely, the organism finds its way into the bloodstream causing severe bacteremia. So far, very few cases of Neisseria bacteremia have been reported. We report a case of a 78-year-old male, post-splenectomy, who presented with high fever, cough and shortness of breath. The patient was initially managed for septic shock with fluid resuscitations, vasopressors and broad-spectrum antibiotics. Later, the blood cultures grew gram-negative coccobacilli, Neisseria cinerea. The patient was successfully treated with intravenous ceftriaxone. This is the first case ever of Neisseria cinerea bacteremia in a post-splenectomy patient and ninth case overall. This case illustrates that the physicians should maintain heightened awareness for Neisseria cinerea bacteremia in post-splenectomy patients.

## Introduction

Neisseria cinerea is a non-pathogenic, gram-negative, catalase-positive and oxidase-positive diplococci [1]. It is an asaccharolytic commensal Neisseria species which usually resides in the upper respiratory tract but sometimes finds its way to bloodstream causing a life-threatening infection. Most of the patients reported with Neisseria cinerea bacteremia have some form of immunodeficiency, while a few were completely healthy. Identifying Neisseria cinerea is a challenge for microbiologists as it shares both biochemical and morphological characteristics with Neisseria gonorrhea and Branhamella catarrhalis [1]. We report first ever case of Neisseria cinerea bacteremia in a post-splenectomy patient rather than the usual encapsulated organisms.

## Case presentation

**Figure 1:**
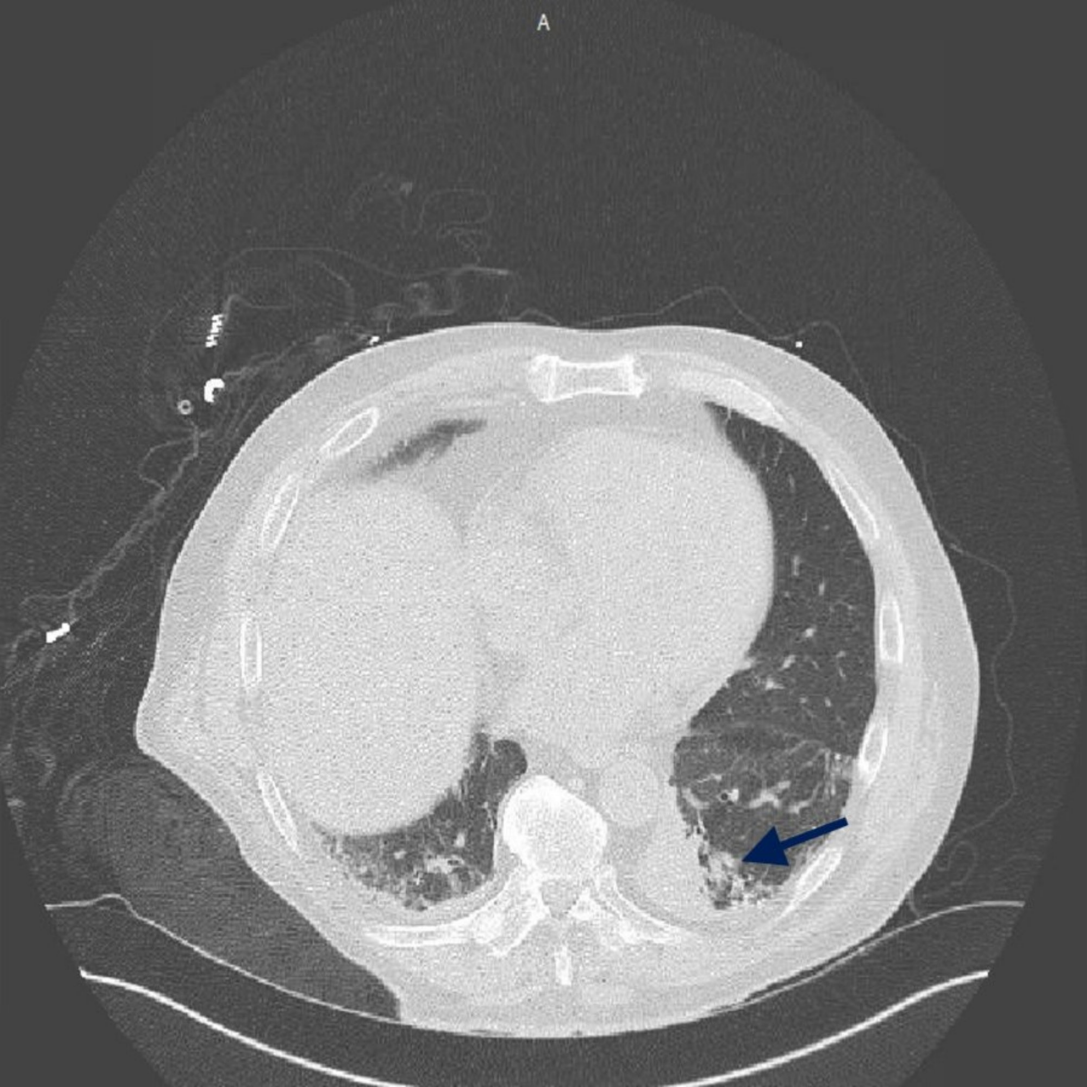
Computed tomography scan of the chest showing consolidation within medial portion of the left lower lobe (blue arrow).

A 78-year-old male presented to the emergency room with complains of high fever, and non-bloody non-bilious vomiting. This was associated with a non-productive cough and dyspnea. He had a past medical history of splenectomy following thrombotic thrombocytopenic purpura (TTP) and recurrent pneumonia. On presentation, the patient was febrile to 101.7° F, respiratory rate of 34 breaths per minute and blood pressure of 83/49 mm Hg. Complete blood count showed leukopenia with white cell count of 1.1 x 10^9^ per liter (L), bandemia of 27% and lactic acidosis of 12.3 mmol/l on the venous blood gas. Computed tomography (CT) scan of the chest/abdomen, and pelvis with oral contrast was performed which showed consolidation in the left lower lobe of lung (Figure 1).

The patient was admitted in the medical intensive care unit with the preliminary diagnosis of severe sepsis with septic shock. Sequential organ failure assessment (SOFA) score at the time of admission was 10. Sepsis bundle was initiated and intravenous (IV) crystalloid resuscitation at 30 ml/kg was immediately started, blood cultures were drawn and intravenous broad-spectrum antibiotics were initiated. Due to inadequate response to intravenous fluids, a vasopressor agent (phenylephrine) and stress dose steroid (hydrocortisone) was started. Blood cultures grew gram-negative coccobacilli, Neisseria species which was later confirmed by microbiologist as Neisseria cinerea. Antibiotics were narrowed down to IV ceftriaxone 2 gm every 12 hours based on the sensitivity.

During the course of hospitalization, the patient improved clinically and remained hemodynamically stable. Repeat blood cultures did not show any growth and the patient was discharged home after completion of two weeks of intravenous ceftriaxone.

## Discussion

Neisseria cinerea was first identified in 1905 as Micrococcus cinereus by Alexander von Lingelsheim [2]. In 1962, Berger and Paepcke described it to be an asaccharolytic commensal of the human oropharynx which was later described correctly as a commensal of human oropharynx and sometimes of the urogenital tract by Knapp et al. in 1984 [2-3]. N. cinerea is an oxidase-positive and catalase-positive, gram-negative diplococci. The bacteria share many morphological and biochemical similarities with Neisseria gonorrhea and Branhamella catarrhalis. Neisseria cinerea is able to reduce glucose like N. gonorrhea but not able to utilize it for energy, making it asaccharolytic. Amino acids like cysteine, cystine, proline and arginine are required for its growth. Unlike Neisseria gonorrhea, N. cinerea can grow on mediums like Mueller-Hinton agar and trypticase soy agar, and is not resistant to colistin [4].

As described earlier, Neisseria cinerea normally colonizes human oropharynx but very rarely enters the bloodstream. All the cases of N. cinerea bacteremia reported to date are described in Table 1 [1, 4-9].

**Table 1:** Table listing all reported cases of Neisseria cinerea.

Patient	Age (years)	Comorbidities	Risk of infection	Additional infection	Antibiotics given	Outcome	Reference
1.	59	Myelodysplastic syndrome	Chemotherapy	None	Imipenem for 14 days	Died	Zhu et al. [1]
2.	58	Multiple sclerosis - Severe Trigeminal neuralgia	Percutaneous glycerol rhizotomy	Meningitis	Ceftriaxone for seven days	Survived	Von Kietzell et al. [5]
3.	47	End-stage renal disease (ESRD) on hemodialysis	Coughing onto arteriovenous graft site	None	Ciprofloxacin	Survived	Johnson et al. [6]
4.	46	Alcohol abuse	None	Acute abdomen with bacteremia	Tobramycin, clindamycin, vancomycin, piperacillin, cefoxiti, ampicillin, amikacin and cefotaxime	Died	Southern & Kutscher [7]
5.	2.5	Frequent upper respiratory tract infections	None	Otitis media and pneumonia	Amoxicillin for 10 days	Survived	Southern & Kutscher [7]
6.	34	Intravenous drug use	Intravenous drug use	Tricuspid valve endocarditis	Co-amoxiclav, ceftriaxone and amoxicillin	Survived	Benes et al. [8]
7.	17	None	Facial trauma with laxation of two teeth	Meningitis	Cefotaxime	Survived	Kirchgesner et al. [9]
8.	38	Postpartum Hemolytic-uremic Syndrome (HUS) causing ESRD	Eculizumab	None	Cefepime	Survived	Walsh et al. [4]
9.	78	Post splenectomy and Thrombotic Thrombocytopenic purpura	Post splenectomy	Left lower lobe pneumonia	Ceftriaxone	Survived	Current case

Our case is the first case of a patient who had undergone splenectomy, who developed Neisseria cinerea bacteremia reported in the literature. Usually, in such patients, encapsulated organisms such as Streptococcus pneumoniae, Haemophilus influenzae or Neisseria meningitidis are the common causes of infection. Immune deficiency was common in most of the previously described cases, and can be considered as the most vital cause leading to bacteremia. Recent reports have demonstrated that despite the use of appropriate vaccination, these immunocompromised patients are still at increased risk of infection with Neisseria bacteremia [10].

## Conclusions

By reporting this case we recommend that Neisseria cinerea bacteremia should be considered in the differential diagnosis of post-splenectomy patients causing infection. A clear understanding of how Neisseria cinerea, an unusual non-encapsulated organism, causes overwhelming sepsis in a post-splenectomy patient, needs further workup. One should also be very cautious in properly isolating Neisseria cinerea due to rarity and similarities with N. gonorrhea.
